# Supplementary figures and images for: Reproducible Propagation of Species-Rich Soil Bacterial Communities Suggests Robust Underlying Deterministic Principles of Community Formation
Source: mSystems. 2022 Mar 30;7(2):e00160-22. doi: 10.1128/msystems.00160-22 (PMC9040596; doi:10.1128/msystems.00160-22)

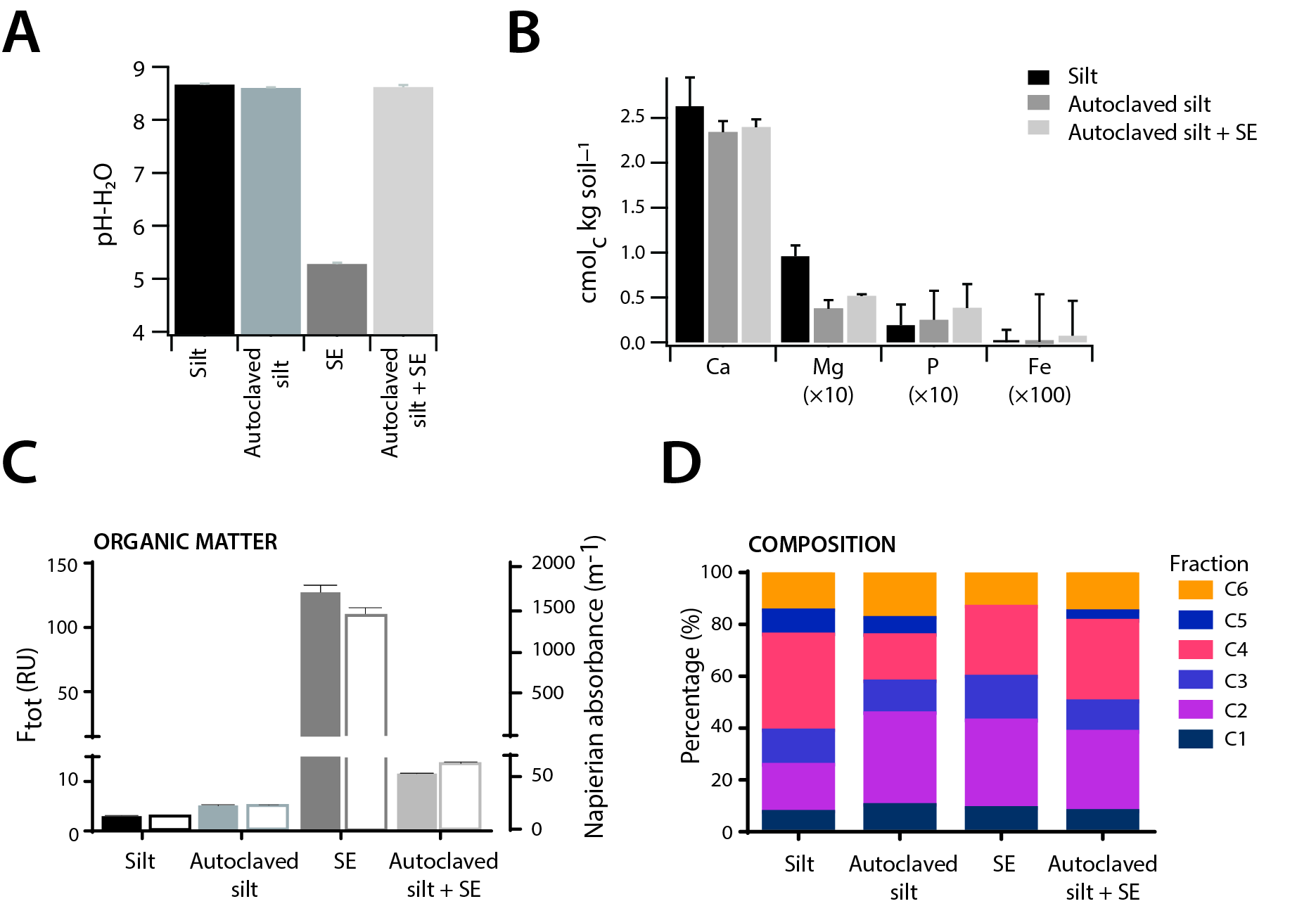

Supplement: FIG S1 [file msystems.00160-22-sf001.tif]

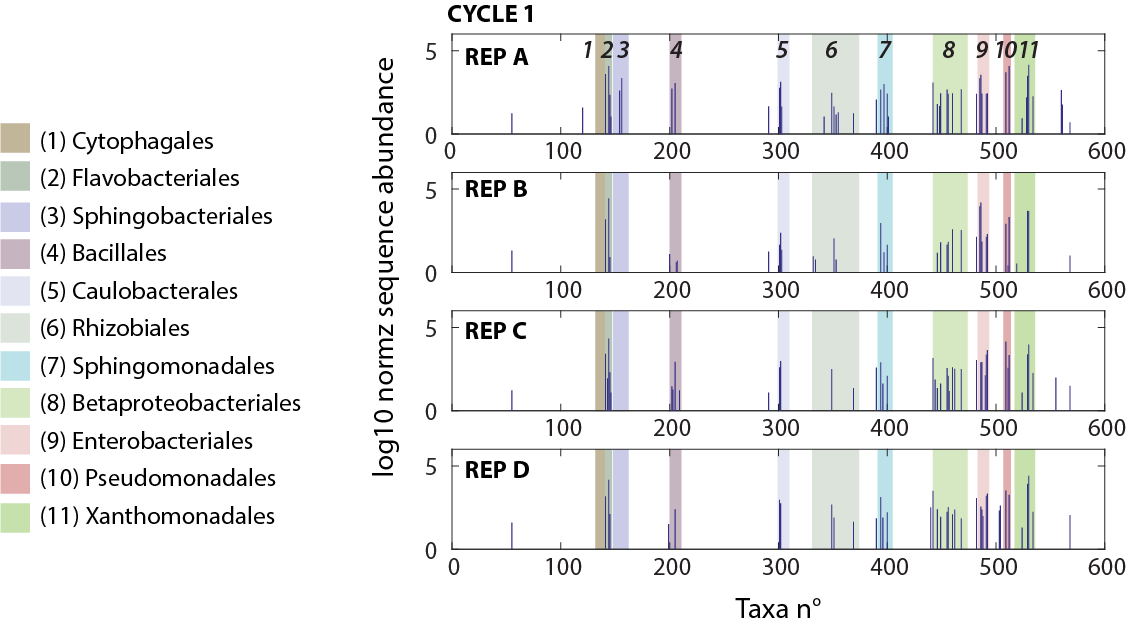

Supplement: FIG S2 [file msystems.00160-22-sf002.tif]

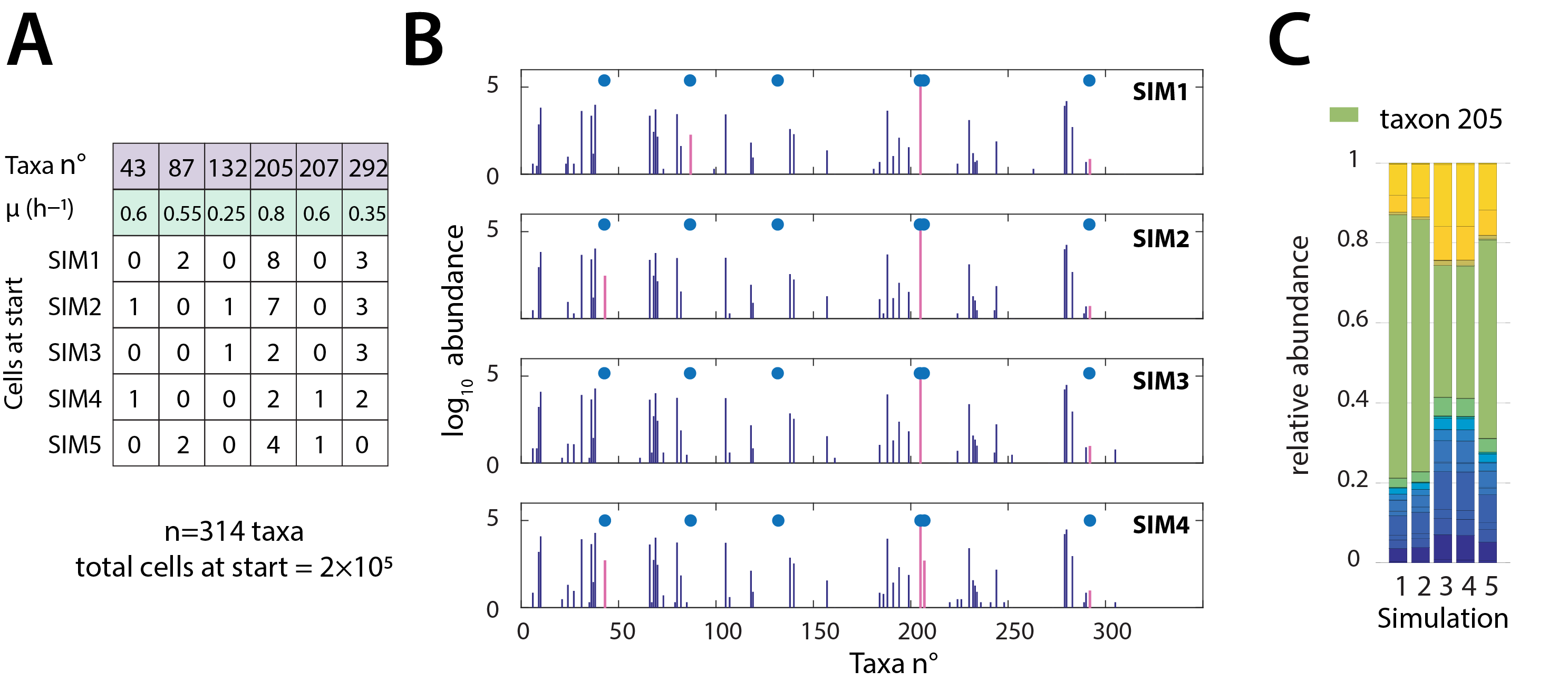

Supplement: FIG S3 [file msystems.00160-22-sf003.tif]

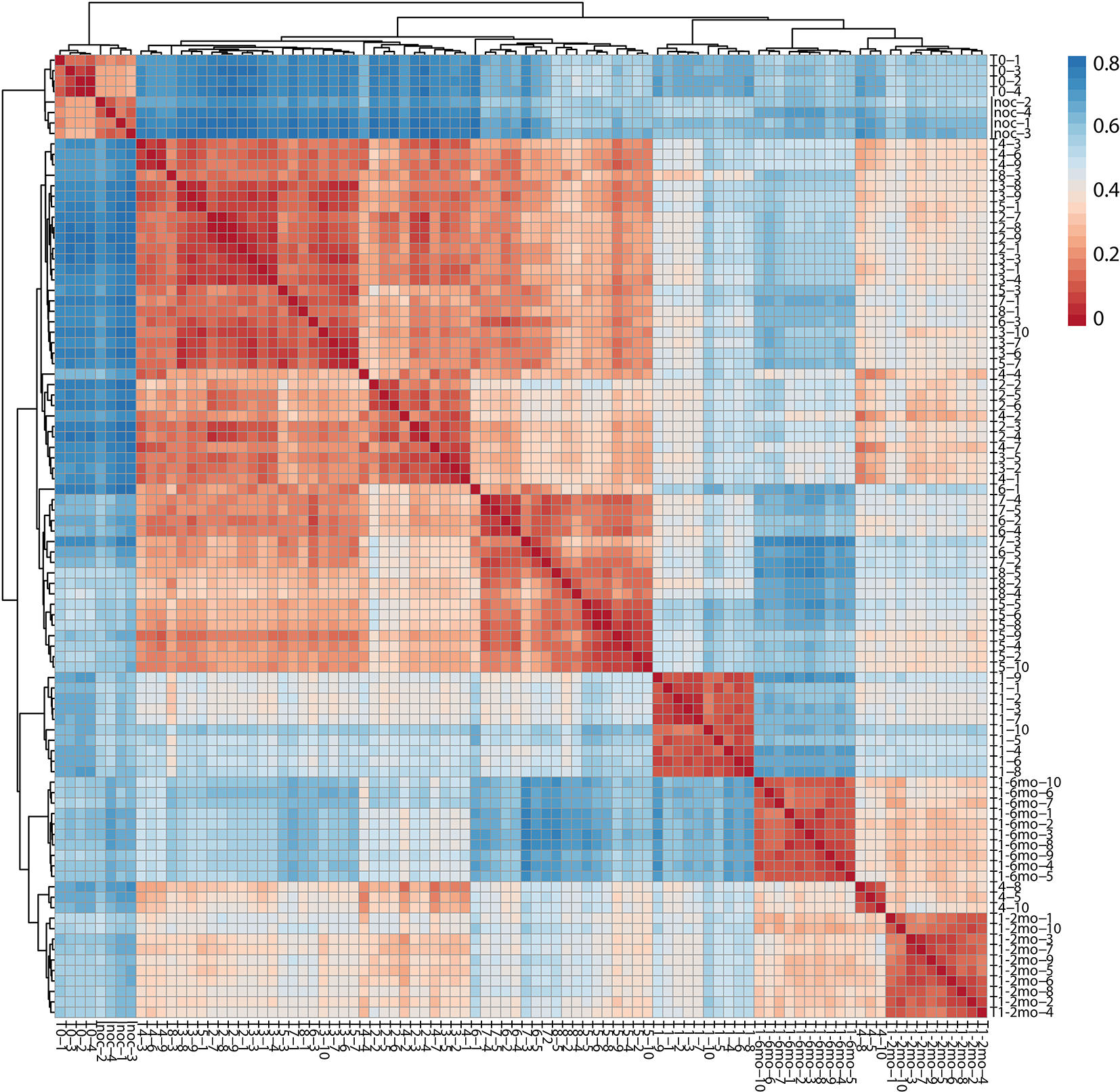

Supplement: FIG S4 [file msystems.00160-22-sf004.tif]

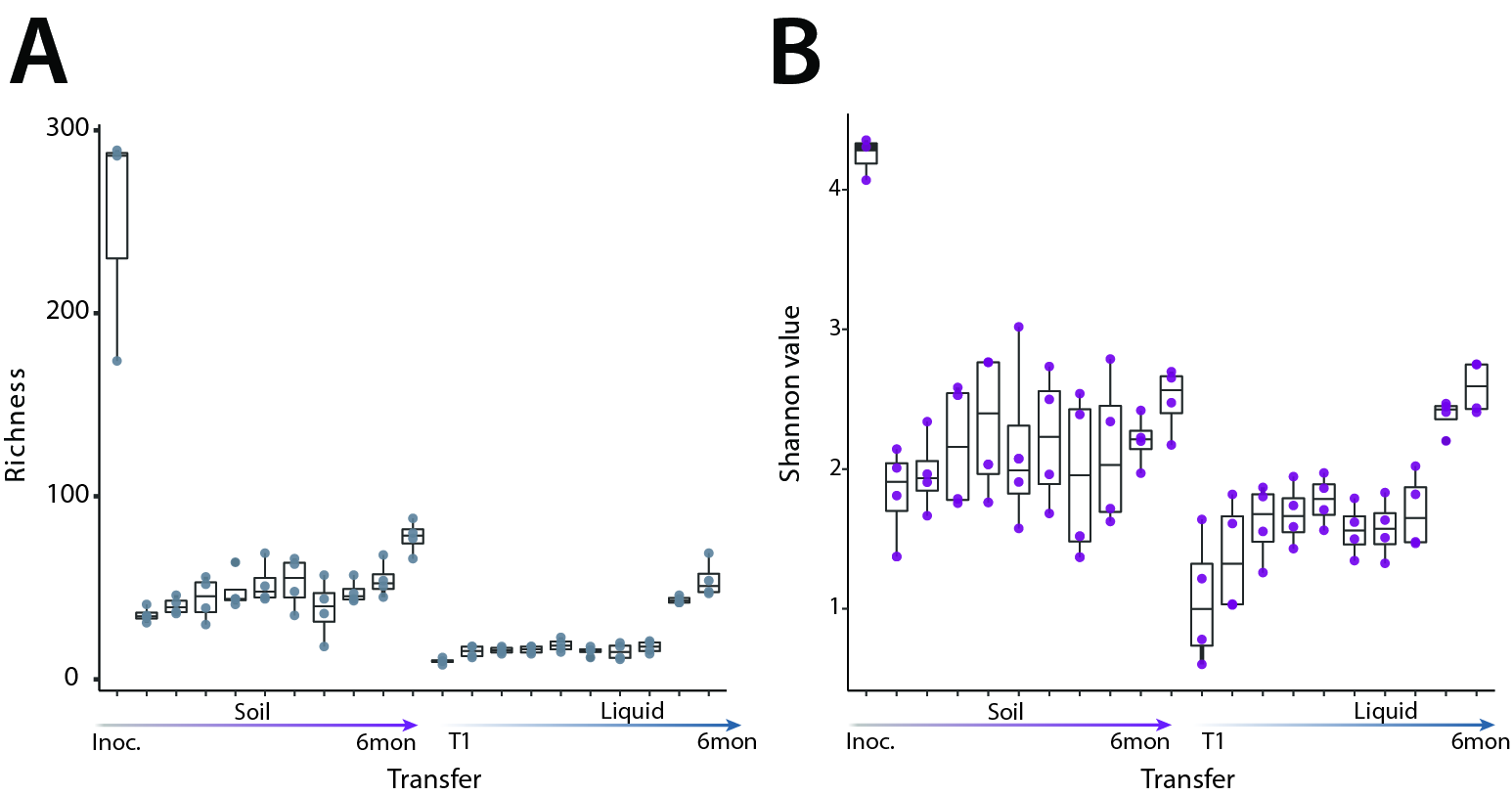

Supplement: FIG S5 [file msystems.00160-22-sf005.tif]

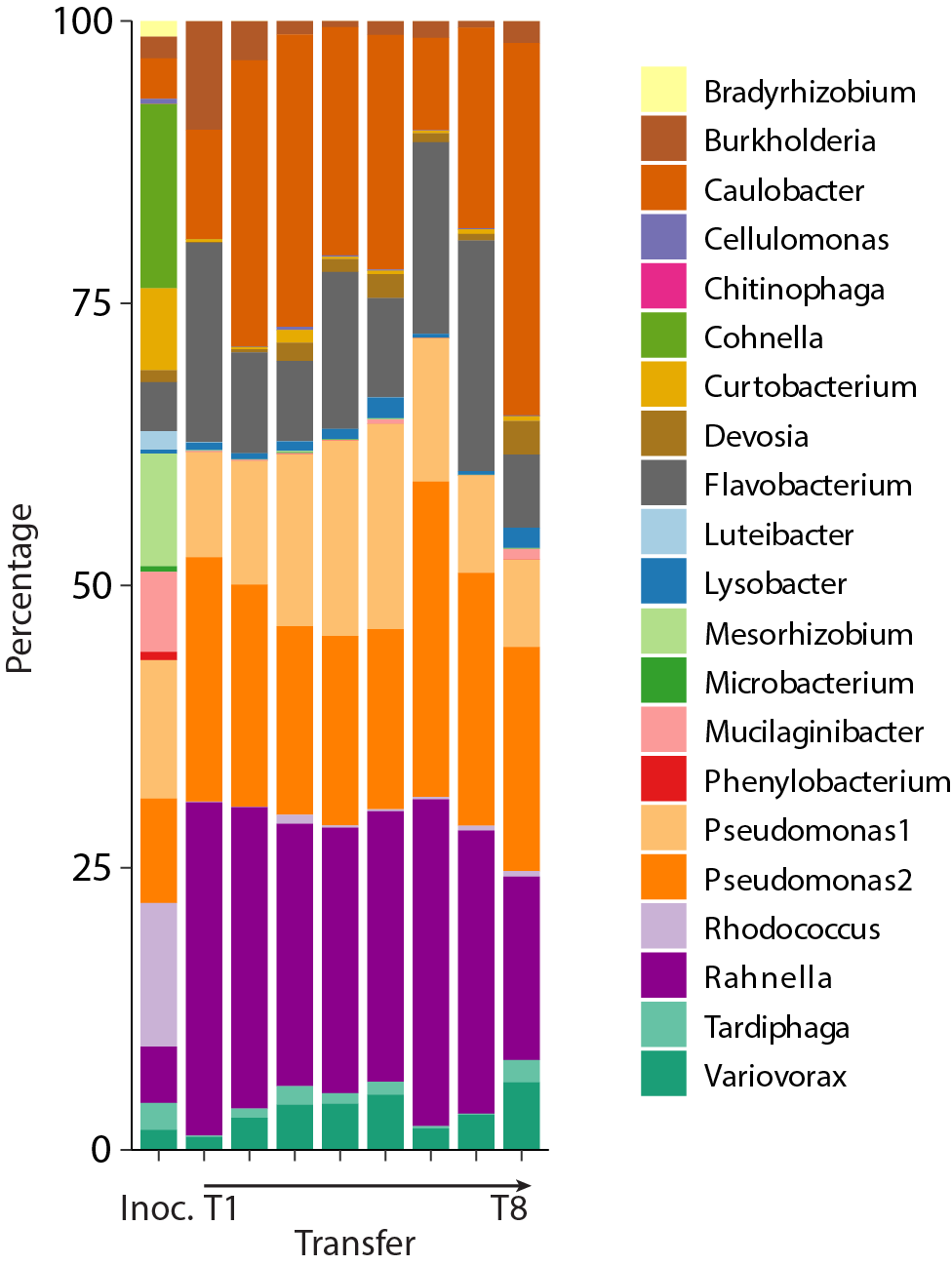

Supplement: FIG S6 [file msystems.00160-22-sf006.tif]

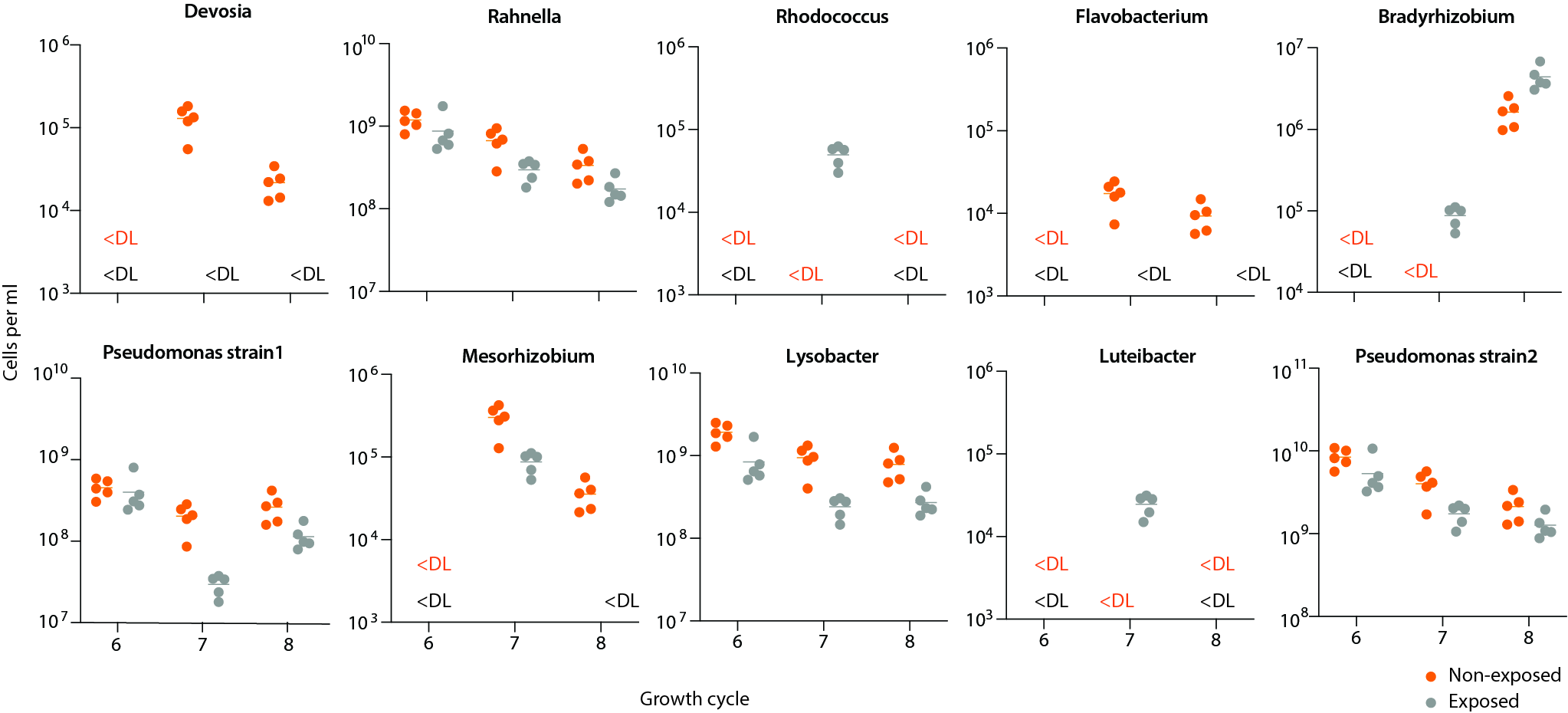

Supplement: FIG S7 [file msystems.00160-22-sf007.tif]
