# Supplementary material for: Reproducible Propagation of Species-Rich Soil Bacterial Communities Suggests Robust Underlying Deterministic Principles of Community Formation
Source: mSystems. 2022 Mar 30;7(2):e00160-22. doi: 10.1128/msystems.00160-22 (PMC9040596; doi:10.1128/msystems.00160-22)
Supplement: TABLE S1 [file msystems.00160-22-st001.docx]

| **Sample** | **PC**  **[%]** | **RC**  **[%]** | **TOC**  **[%]** | **MINC**  **[%]** | **HI**  **[mg HC/g TOC]** | **OI**  **[mg CO_2_/g TOC]** | **NO_3_-N [mg/kg]** | **NH_4_-N [mg/kg]** | **Total-N [%]** |
| --- | --- | --- | --- | --- | --- | --- | --- | --- | --- |
| Silt | 0.04 | 0.10 | 0.13 | 2.75 | 151 | 563 | nd | nd | nd |
| Autoclaved Silt | 0.04 | 0.11 | 0.15 | 3.62 | 161 | 426 | nd | nd | nd |
| Autoclaved Silt + SE | 0.03 | 0.12 | 0.15 | 3.06 | 116 | 379 | <0.01 | 2.31 | 0.03 |

PC, pyrolyzable carbon fraction; RC, residual carbon fraction; MINC, mineral carbon fraction; TOC, total organic carbon; HI, hydrogen index; OI, oxygen index; nd, non-determined.
