# Supplementary material for: Reproducible Propagation of Species-Rich Soil Bacterial Communities Suggests Robust Underlying Deterministic Principles of Community Formation
Source: mSystems. 2022 Mar 30;7(2):e00160-22. doi: 10.1128/msystems.00160-22 (PMC9040596; doi:10.1128/msystems.00160-22)
Supplement: TABLE S2 [file msystems.00160-22-st002.docx]

| **Isolate No.** | **slv_last taxa** | **phyla;class** |
| --- | --- | --- |
| 1 | Actinobacteria | Proteobacteria;Gammaproteobacteria |
| 2 | Agromyces | Actinobacteria;Actinobacteria |
| 3 | Agromyces | Actinobacteria;Actinobacteria |
| 4 | Agromyces | Actinobacteria;Actinobacteria |
| 5 | Agromyces | Actinobacteria;Actinobacteria |
| 6 | Agromyces | Actinobacteria;Actinobacteria |
| 7 | Agromyces | Actinobacteria;Actinobacteria |
| 8 | Agromyces | Actinobacteria;Actinobacteria |
| 9 | Agromyces | Actinobacteria;Actinobacteria |
| 10 | Allorhizobium-Neorhizobium-Pararhizobium-Rhizobium | Proteobacteria;Alphaproteobacteria |
| 11 | Allorhizobium-Neorhizobium-Pararhizobium-Rhizobium | Proteobacteria;Alphaproteobacteria |
| 12 | Allorhizobium-Neorhizobium-Pararhizobium-Rhizobium; | Proteobacteria;Alphaproteobacteria |
| 13 | Altererythrobacter | Proteobacteria;Alphaproteobacteria |
| 14 | Aminobacter | Proteobacteria;Alphaproteobacteria |
| 15 | Angustibacter | Actinobacteria;Actinobacteria |
| 16 | Angustibacter | Actinobacteria;Actinobacteria |
| 17 | Angustibacter | Actinobacteria;Actinobacteria |
| 18 | Angustibacter | Actinobacteria;Actinobacteria |
| 19 | Angustibacter | Actinobacteria;Actinobacteria |
| 20 | Bosea | Proteobacteria;Alphaproteobacteria |
| 21 | Bosea | Proteobacteria;Alphaproteobacteria |
| 22 | Bosea | Proteobacteria;Alphaproteobacteria |
| 23 | Bradyrhizobium | Proteobacteria;Alphaproteobacteria |
| 24 | Bradyrhizobium | Proteobacteria;Alphaproteobacteria |
| 25 | Bradyrhizobium | Proteobacteria;Alphaproteobacteria |
| 26 | Bradyrhizobium | Proteobacteria;Alphaproteobacteria |
| 27 | Bradyrhizobium | Proteobacteria;Alphaproteobacteria |
| 28 | Bradyrhizobium | Proteobacteria;Alphaproteobacteria |
| 29 | Bradyrhizobium | Proteobacteria;Alphaproteobacteria |
| 30 | Bradyrhizobium | Proteobacteria;Alphaproteobacteria |
| 31 | Bradyrhizobium | Proteobacteria;Alphaproteobacteria |
| 32 | Bradyrhizobium | Proteobacteria;Alphaproteobacteria |
| 33 | Bradyrhizobium | Proteobacteria;Alphaproteobacteria |
| 34 | Bradyrhizobium | Proteobacteria;Alphaproteobacteria |
| 35 | Bradyrhizobium | Proteobacteria;Alphaproteobacteria |
| 36 | Bradyrhizobium | Proteobacteria;Alphaproteobacteria |
| 37 | Burkholderia-Caballeronia-Paraburkholderia | Proteobacteria;Gammaproteobacteria |
| 38 | Burkholderia-Caballeronia-Paraburkholderia | Proteobacteria;Betaproteobacteria |
| 39 | Burkholderia-Caballeronia-Paraburkholderia | Proteobacteria;Betaproteobacteria |
| 40 | Burkholderia-Caballeronia-Paraburkholderia | Proteobacteria;Betaproteobacteria |
| 41 | Burkholderia-Caballeronia-Paraburkholderia | Proteobacteria;Betaproteobacteria |
| 42 | Burkholderia-Caballeronia-Paraburkholderia | Proteobacteria;Betaproteobacteria |
| 43 | Burkholderia-Caballeronia-Paraburkholderia | Proteobacteria;Betaproteobacteria |
| 44 | Burkholderia-Caballeronia-Paraburkholderia | Proteobacteria;Betaproteobacteria |
| 45 | Burkholderia-Caballeronia-Paraburkholderia | Proteobacteria;Betaproteobacteria |
| 46 | Burkholderia-Caballeronia-Paraburkholderia | Proteobacteria;Betaproteobacteria |
| 47 | Burkholderia-Caballeronia-Paraburkholderia | Proteobacteria;Betaproteobacteria |
| 48 | Burkholderia-Caballeronia-Paraburkholderia | Proteobacteria;Betaproteobacteria |
| 49 | Burkholderia-Caballeronia-Paraburkholderia | Proteobacteria;Betaproteobacteria |
| 50 | Burkholderia-Caballeronia-Paraburkholderia | Proteobacteria;Betaproteobacteria |
| 51 | Burkholderiaceae | Proteobacteria;Gammaproteobacteria |
| 52 | Burkholderiaceae | Proteobacteria;Gammaproteobacteria |
| 53 | Caulobacter | Proteobacteria;Alphaproteobacteria |
| 54 | Caulobacter | Proteobacteria;Alphaproteobacteria |
| 55 | Caulobacter | Proteobacteria;Alphaproteobacteria |
| 56 | Caulobacter | Proteobacteria;Alphaproteobacteria |
| 57 | Caulobacter | Proteobacteria;Alphaproteobacteria |
| 58 | Cellulomonas | Actinobacteria;Actinobacteria |
| 59 | Cellulomonas | Actinobacteria;Actinobacteria |
| 60 | Cellulomonas | Actinobacteria;Actinobacteria |
| 61 | Cellulomonas | Actinobacteria;Actinobacteria |
| 62 | Cellulomonas | Actinobacteria;Actinobacteria |
| 63 | Chitinophaga | Bacteroidetes;Bacteroidia |
| 64 | Chitinophaga | Bacteroidetes;Bacteroidia |
| 65 | Cohnella | Firmicutes;Bacilli |
| 66 | Curtobacterium | Actinobacteria;Actinobacteria |
| 67 | Curtobacterium | Actinobacteria;Actinobacteria |
| 68 | Devosia | Proteobacteria;Alphaproteobacteria |
| 69 | Dyella | Proteobacteria;Gammaproteobacteria |
| 70 | Dyella | Proteobacteria;Gammaproteobacteria |
| 71 | Ensifer | Proteobacteria;Alphaproteobacteria |
| 72 | Ensifer | Proteobacteria;Alphaproteobacteria |
| 73 | Enterobacteriaceae | Proteobacteria;Gammaproteobacteria |
| 74 | Enterobacteriaceae | Proteobacteria;Gammaproteobacteria |
| 75 | Enterobacteriaceae | Proteobacteria;Gammaproteobacteria |
| 76 | Enterobacteriaceae | Proteobacteria;Gammaproteobacteria |
| 77 | Enterobacteriaceae | Proteobacteria;Gammaproteobacteria |
| 78 | Enterobacteriaceae | Proteobacteria;Gammaproteobacteria |
| 79 | Flavobacterium | Bacteroidetes;Bacteroidia |
| 80 | Flavobacterium | Bacteroidetes;Bacteroidia |
| 81 | Flavobacterium | Bacteroidetes;Bacteroidia |
| 82 | Frondihabitans | Actinobacteria;Actinobacteria |
| 83 | Frondihabitans | Actinobacteria;Actinobacteria |
| 84 | Kluyvera | Proteobacteria;Gammaproteobacteria |
| 85 | Labrys | Proteobacteria;Alphaproteobacteria |
| 86 | Labrys | Proteobacteria;Alphaproteobacteria |
| 87 | Labrys | Proteobacteria;Alphaproteobacteria |
| 88 | Leifsonia | Actinobacteria;Actinobacteria |
| 89 | Leifsonia | Actinobacteria;Actinobacteria |
| 90 | Luteibacter | Proteobacteria;Gammaproteobacteria |
| 91 | Luteibacter | Proteobacteria;Gammaproteobacteria |
| 92 | Lysobacter | Proteobacteria;Gammaproteobacteria |
| 93 | Mesorhizobium | Proteobacteria;Alphaproteobacteria |
| 94 | Mesorhizobium | Proteobacteria;Alphaproteobacteria |
| 95 | Mesorhizobium | Proteobacteria;Alphaproteobacteria |
| 96 | Mesorhizobium | Proteobacteria;Alphaproteobacteria |
| 97 | Mesorhizobium | Proteobacteria;Alphaproteobacteria |
| 98 | Mesorhizobium | Proteobacteria;Alphaproteobacteria |
| 99 | Mesorhizobium | Proteobacteria;Alphaproteobacteria |
| 100 | Mesorhizobium | Proteobacteria;Alphaproteobacteria |
| 101 | Methylobacterium | Proteobacteria;Alphaproteobacteria |
| 102 | Microbacteriaceae | Actinobacteria;Actinobacteria |
| 103 | Microbacterium | Actinobacteria;Actinobacteria |
| 104 | Microbacterium | Actinobacteria;Actinobacteria |
| 105 | Microbacterium | Actinobacteria;Actinobacteria |
| 106 | Microbacterium | Actinobacteria;Actinobacteria |
| 107 | Microbacterium | Actinobacteria;Actinobacteria |
| 108 | Microbacterium | Actinobacteria;Actinobacteria |
| 109 | Microbacterium | Actinobacteria;Actinobacteria |
| 110 | Microbacterium | Actinobacteria;Actinobacteria |
| 111 | Microbacterium | Actinobacteria;Actinobacteria |
| 112 | Microbacterium | Actinobacteria;Actinobacteria |
| 113 | Mucilaginibacter | Bacteroidetes;Bacteroidia |
| 114 | Mucilaginibacter | Bacteroidetes;Bacteroidia |
| 115 | Mucilaginibacter | Bacteroidetes;Bacteroidia |
| 116 | Mumia | Actinobacteria;Actinobacteria |
| 117 | Mycobacterium | Actinobacteria;Actinobacteria |
| 118 | Mycobacterium | Actinobacteria;Actinobacteria |
| 119 | Mycobacterium | Actinobacteria;Actinobacteria |
| 120 | Nocardioides | Actinobacteria;Actinobacteria |
| 121 | Nocardioides | Actinobacteria;Actinobacteria |
| 122 | Nocardioides | Actinobacteria;Actinobacteria |
| 123 | Nocardioides | Actinobacteria;Actinobacteria |
| 124 | Nocardioides | Actinobacteria;Actinobacteria |
| 125 | Nocardioides | Actinobacteria;Actinobacteria |
| 126 | Nocardioides | Actinobacteria;Actinobacteria |
| 127 | Nocardioides | Actinobacteria;Actinobacteria |
| 128 | Nocardioides | Actinobacteria;Actinobacteria |
| 129 | Nocardioides | Actinobacteria;Actinobacteria |
| 130 | Nocardioides | Actinobacteria;Actinobacteria |
| 131 | Nocardioides | Actinobacteria;Actinobacteria |
| 132 | Nonomuraea | Actinobacteria;Actinobacteria |
| 133 | Nonomuraea | Actinobacteria;Actinobacteria |
| 134 | Phenylobacterium | Proteobacteria;Alphaproteobacteria |
| 135 | Phycicoccus | Actinobacteria;Actinobacteria |
| 136 | Phycicoccus | Actinobacteria;Actinobacteria |
| 137 | Pseudomonas | Proteobacteria;Gammaproteobacteria |
| 138 | Pseudomonas | Proteobacteria;Gammaproteobacteria |
| 139 | Pseudomonas | Proteobacteria;Gammaproteobacteria |
| 140 | Pseudomonas | Proteobacteria;Gammaproteobacteria |
| 141 | Pseudomonas | Proteobacteria;Gammaproteobacteria |
| 142 | Pseudoxanthomonas | Proteobacteria;Gammaproteobacteria |
| 143 | Pseudoxanthomonas | Proteobacteria;Gammaproteobacteria |
| 144 | Rhizobacter | Proteobacteria;Gammaproteobacteria |
| 145 | Rhizobacter | Proteobacteria;Gammaproteobacteria |
| 146 | Rhizobacter | Proteobacteria;Gammaproteobacteria |
| 147 | Rhizobiaceae | Proteobacteria;Alphaproteobacteria |
| 148 | Rhizobiaceae | Proteobacteria;Alphaproteobacteria |
| 149 | Rhizobiales | Proteobacteria;Alphaproteobacteria |
| 150 | Rhodococcus | Actinobacteria;Actinobacteria |
| 151 | Rhodococcus; | Actinobacteria;Actinobacteria |
| 152 | Roseateles | Proteobacteria;Gammaproteobacteria |
| 153 | Serratia | Proteobacteria;Gammaproteobacteria |
| 154 | Serratia | Proteobacteria;Gammaproteobacteria |
| 155 | Sphingomonadaceae;uncultured | Proteobacteria;Alphaproteobacteria |
| 156 | Sphingomonas | Proteobacteria;Alphaproteobacteria |
| 157 | Sphingomonas | Proteobacteria;Alphaproteobacteria |
| 158 | Sphingopyxis | Proteobacteria;Alphaproteobacteria |
| 159 | Sphingopyxis | Proteobacteria;Alphaproteobacteria |
| 160 | Stenotrophomonas | Proteobacteria;Gammaproteobacteria |
| 161 | Streptomyces | Actinobacteria;Actinobacteria |
| 162 | Streptomyces | Actinobacteria;Actinobacteria |
| 163 | Tardiphaga | Proteobacteria;Alphaproteobacteria |
| 164 | Variovorax | Proteobacteria;Gammaproteobacteria |
| 165 | Xanthobacteraceae | Proteobacteria;Alphaproteobacteria |
| 166 | Xanthobacteraceae;uncultured | Proteobacteria;Alphaproteobacteria |
| 167 | Unclassified; | Unclassified |
| 168 | Unclassified; | Unclassified |
| 169 | Unclassified; | Unclassified |
